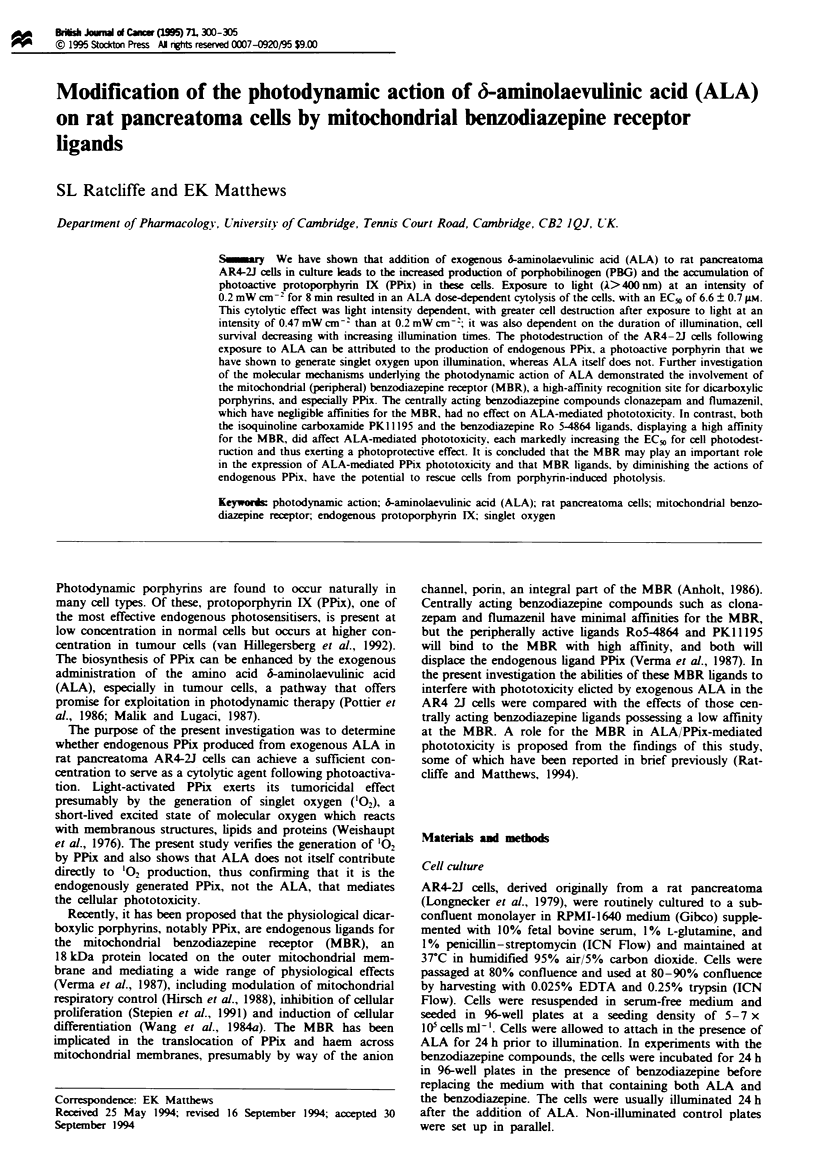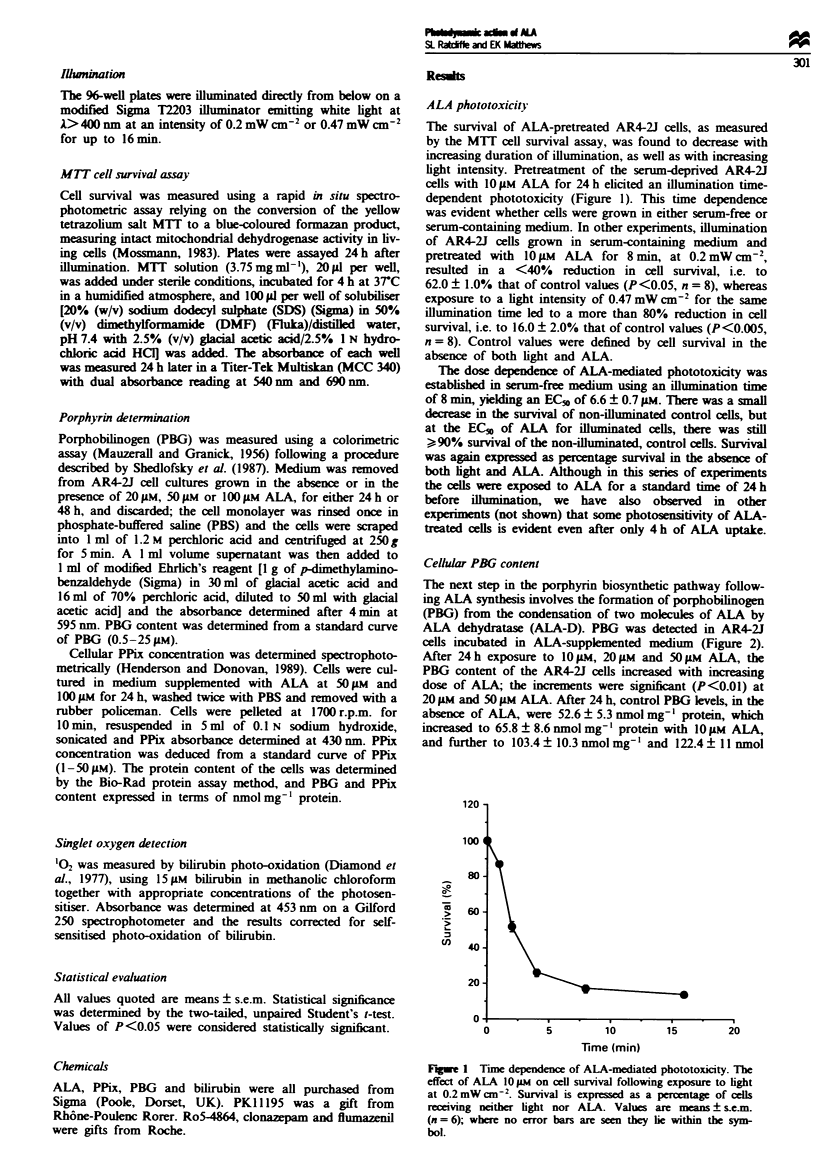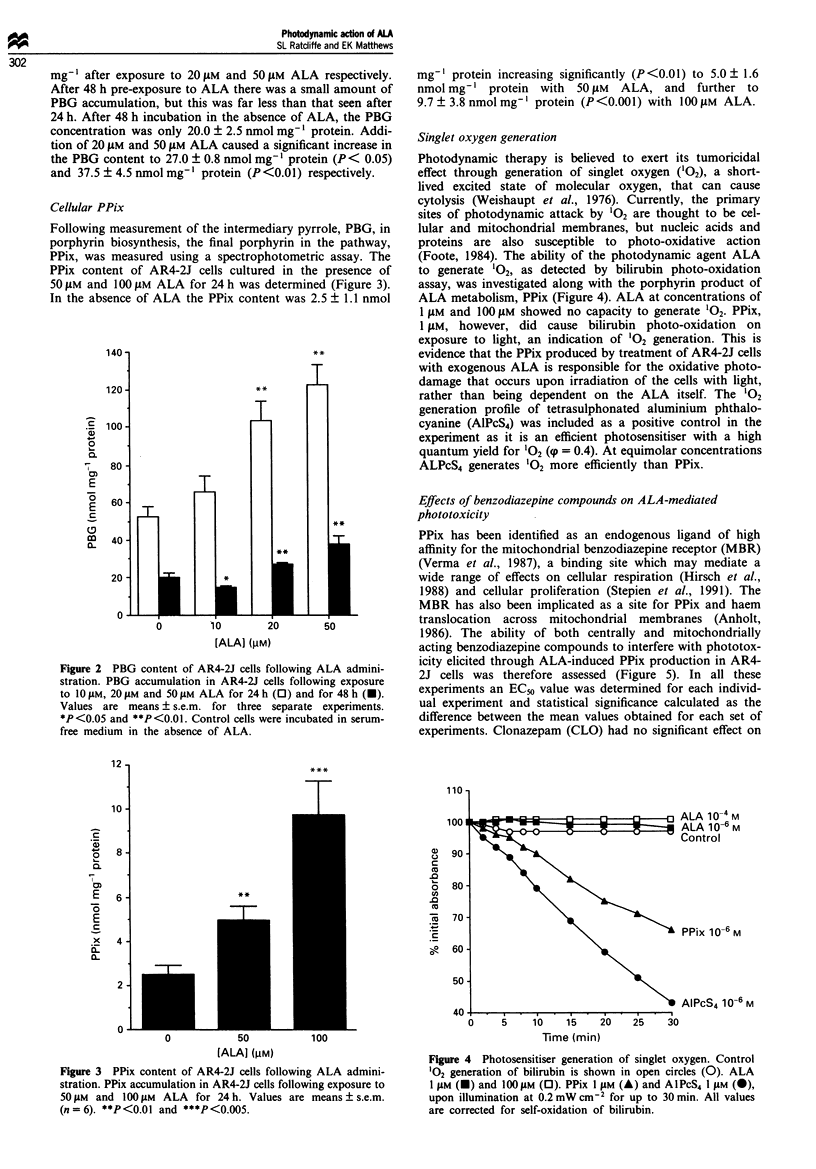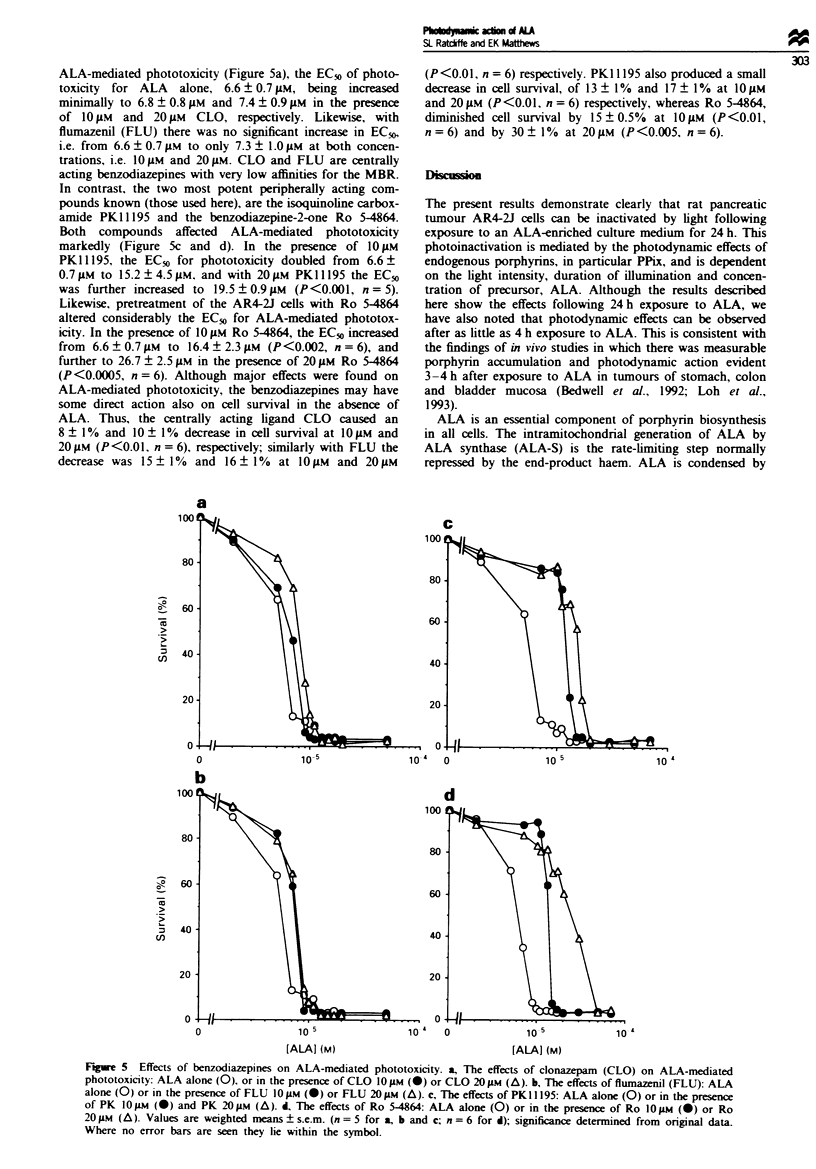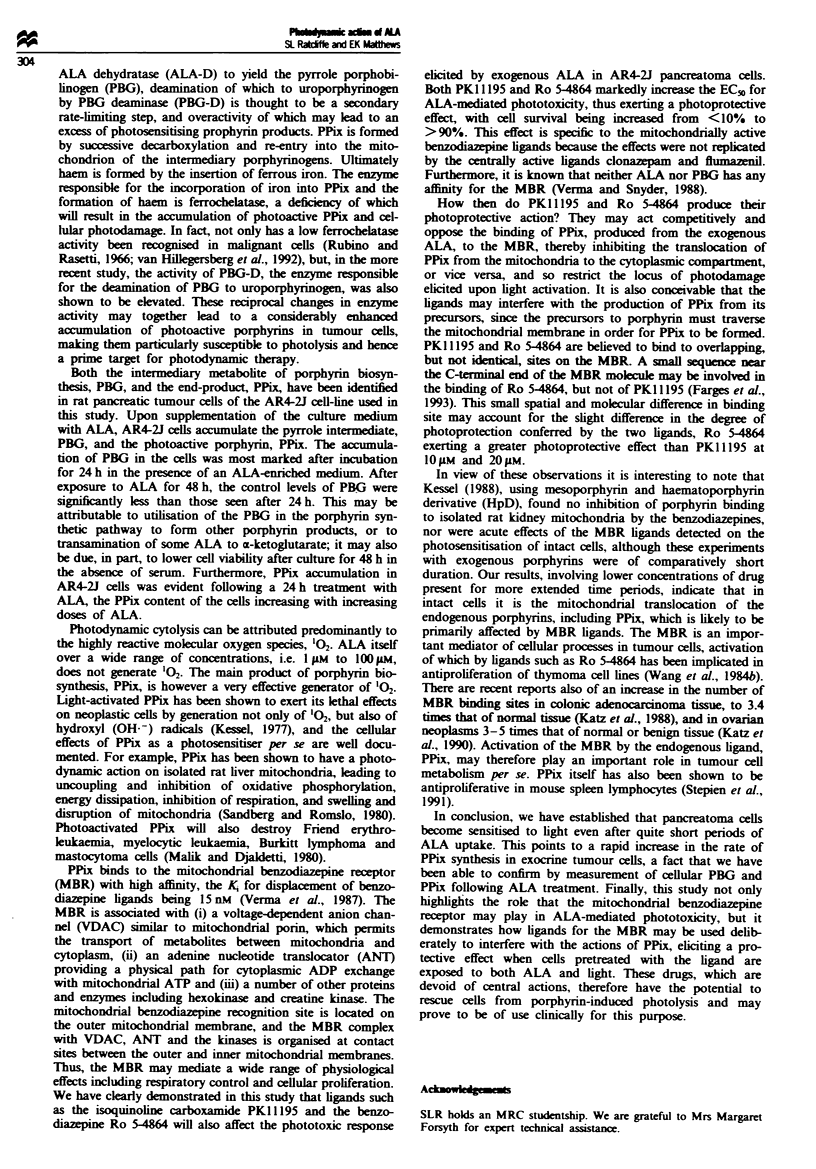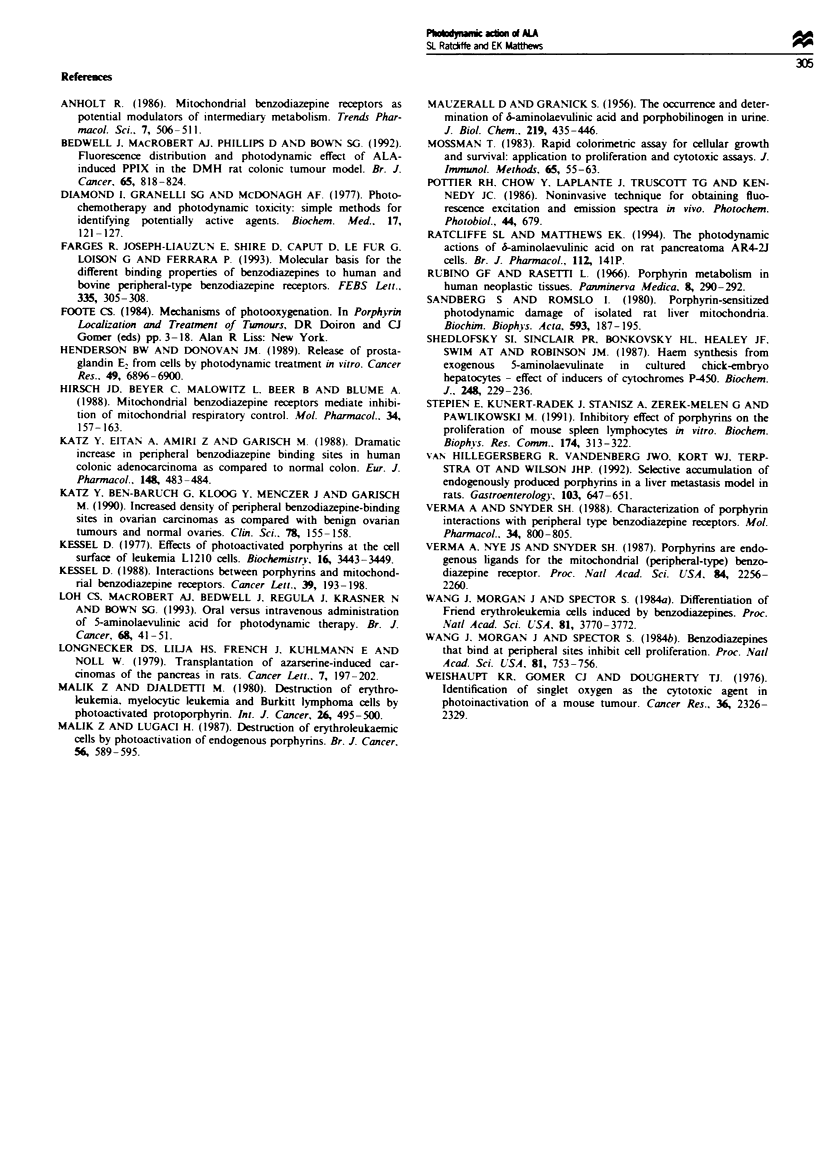# Modification of the photodynamic action of delta-aminolaevulinic acid (ALA) on rat pancreatoma cells by mitochondrial benzodiazepine receptor ligands.

**DOI:** 10.1038/bjc.1995.60

**Published:** 1995-02

**Authors:** S. L. Ratcliffe, E. K. Matthews

**Affiliations:** Department of Pharmacology, University of Cambridge, UK.

## Abstract

We have shown that addition of exogenous delta-aminolaevulinic acid (ALA) to rat pancreatoma AR4-2J cells in culture leads to the increased production of porphobilinogen (PBG) and the accumulation of photoactive protoporphyrin IX (PPix) in these cells. Exposure to light (lambda > 400 nm) at an intensity of 0.2 mW cm-2 for 8 min resulted in an ALA dose-dependent cytolysis of the cells, with an EC50 of 6.6 +/- 0.7 microM. This cytolytic effect was light intensity dependent, with greater cell destruction after exposure to light at an intensity of 0.47 mW cm-2 than at 0.2 mW cm-2; it was also dependent on the duration of illumination, cell survival decreasing with increasing illumination times. The photodestruction of the AR4-2J cells following exposure to ALA can be attributed to the production of endogenous PPix, a photoactive porphyrin that we have shown to generate singlet oxygen upon illumination, whereas ALA itself does not. Further investigation of the molecular mechanisms underlying the photodynamic action of ALA demonstrated the involvement of the mitochondrial (peripheral) benzodiazepine receptor (MBR), a high-affinity recognition site for dicarboxylic porphyrins, and especially PPix. The centrally acting benzodiazepine compounds clonazepam and flumazenil, which have negligible affinities for the MBR, had no effect on ALA-mediated phototoxicity. In contrast, both the isoquinoline carboxamide PK11195 and the benzodiazepine Ro 5-4864 ligands, displaying a high affinity for the MBR, did affect ALA-mediated phototoxicity, each markedly increasing the EC50 for cell photodestruction and thus exerting a photoprotective effect. It is concluded that the MBR may play an important role in the expression of ALA-mediated PPix phototoxicity and that MBR ligands, by diminishing the actions of endogenous PPix, have the potential to rescue cells from porphyrin-induced photolysis.


© 1995 Stockton Press All rights reserved 0007-0920/95 $9.00

### Modification of the photodynamic action of δ-aminolaevulinic acid (ALA) on rat pancreatoma cells by mitochondrial benzodiazepine receptor ligands

SL Ratcliffe and EK Matthews

*Department of Pharmacology, University of Cambridge, Tennis Court Road, Cambridge, CB2 1QJ, UK.*

**Summary** We have shown that addition of exogenous δ-aminolaevulinic acid (ALA) to rat pancreatoma AR4-2J cells in culture leads to the increased production of porphobilinogen (PBG) and the accumulation of photoactive protoporphyrin IX (PPix) in these cells. Exposure to light ($\lambda > 400$ nm) at an intensity of 0.2 mW $cm^{-2}$ for 8 min resulted in an ALA dose-dependent cytolysis of the cells, with an $EC_{50}$ of $6.6 \pm 0.7$ μM. This cytolytic effect was light intensity dependent, with greater cell destruction after exposure to light at an intensity of 0.47 mW $cm^{-2}$ than at 0.2 mW $cm^{-2}$; it was also dependent on the duration of illumination, cell survival decreasing with increasing illumination times. The photodestruction of the AR4-2J cells following exposure to ALA can be attributed to the production of endogenous PPix, a photoactive porphyrin that we have shown to generate singlet oxygen upon illumination, whereas ALA itself does not. Further investigation of the molecular mechanisms underlying the photodynamic action of ALA demonstrated the involvement of the mitochondrial (peripheral) benzodiazepine receptor (MBR), a high-affinity recognition site for dicarboxylic porphyrins, and especially PPix. The centrally acting benzodiazepine compounds clonazepam and flumazenil, which have negligible affinities for the MBR, had no effect on ALA-mediated phototoxicity. In contrast, both the isoquinoline carboxamide PK11195 and the benzodiazepine Ro 5-4864 ligands, displaying a high affinity for the MBR, did affect ALA-mediated phototoxicity, each markedly increasing the $EC_{50}$ for cell photodestruction and thus exerting a photoprotective effect. It is concluded that the MBR may play an important role in the expression of ALA-mediated PPix phototoxicity and that MBR ligands, by diminishing the actions of endogenous PPix, have the potential to rescue cells from porphyrin-induced photolysis.

**Keywords:** photodynamic action; δ-aminolaevulinic acid (ALA); rat pancreatoma cells; mitochondrial benzodiazepine receptor; endogenous protoporphyrin IX; singlet oxygen

Photodynamic porphyrins are found to occur naturally in many cell types. Of these, protoporphyrin IX (PPix), one of the most effective endogenous photosensitisers, is present at low concentration in normal cells but occurs at higher concentration in tumour cells (van Hillegersberg *et al.*, 1992). The biosynthesis of PPix can be enhanced by the exogenous administration of the amino acid δ-aminolaevulinic acid (ALA), especially in tumour cells, a pathway that offers promise for exploitation in photodynamic therapy (Pottier *et al.*, 1986; Malik and Lugaci, 1987).

The purpose of the present investigation was to determine whether endogenous PPix produced from exogenous ALA in rat pancreatoma AR4-2J cells can achieve a sufficient concentration to serve as a cytolytic agent following photoactivation. Light-activated PPix exerts its tumoricidal effect presumably by the generation of singlet oxygen ($^1O_2$), a short-lived excited state of molecular oxygen which reacts with membranous structures, lipids and proteins (Weishaupt *et al.*, 1976). The present study verifies the generation of $^1O_2$ by PPix and also shows that ALA does not itself contribute directly to $^1O_2$ production, thus confirming that it is the endogenously generated PPix, not the ALA, that mediates the cellular phototoxicity.

Recently, it has been proposed that the physiological dicarboxylic porphyrins, notably PPix, are endogenous ligands for the mitochondrial benzodiazepine receptor (MBR), an 18 kDa protein located on the outer mitochondrial membrane and mediating a wide range of physiological effects (Verma *et al.*, 1987), including modulation of mitochondrial respiratory control (Hirsch *et al.*, 1988), inhibition of cellular proliferation (Stepien *et al.*, 1991) and induction of cellular differentiation (Wang *et al.*, 1984*a*). The MBR has been implicated in the translocation of PPix and haem across mitochondrial membranes, presumably by way of the anion channel, porin, an integral part of the MBR (Anholt, 1986). Centrally acting benzodiazepine compounds such as clonazepam and flumazenil have minimal affinities for the MBR, but the peripherally active ligands Ro5-4864 and PK11195 will bind to the MBR with high affinity, and both will displace the endogenous ligand PPix (Verma *et al.*, 1987). In the present investigation the abilities of these MBR ligands to interfere with phototoxicity elicted by exogenous ALA in the AR4 2J cells were compared with the effects of those centrally acting benzodiazepine ligands possessing a low affinity at the MBR. A role for the MBR in ALA PPix-mediated phototoxicity is proposed from the findings of this study, some of which have been reported in brief previously (Ratcliffe and Matthews, 1994).

### Materials and methods

### *Cell culture*

AR4-2J cells, derived originally from a rat pancreatoma (Longnecker *et al.*, 1979), were routinely cultured to a subconfluent monolayer in RPMI-1640 medium (Gibco) supplemented with 10% fetal bovine serum, 1% L-glutamine, and 1% penicillin-streptomycin (ICN Flow) and maintained at 37°C in humidified 95% air 5% carbon dioxide. Cells were passaged at 80% confluence and used at 80-90% confluence by harvesting with 0.025% EDTA and 0.25% trypsin (ICN Flow). Cells were resuspended in serum-free medium and seeded in 96-well plates at a seeding density of $5-7 \times 10^5$ cells $ml^{-1}$. Cells were allowed to attach in the presence of ALA for 24 h prior to illumination. In experiments with the benzodiazepine compounds, the cells were incubated for 24 h in 96-well plates in the presence of benzodiazepine before replacing the medium with that containing both ALA and the benzodiazepine. The cells were usually illuminated 24 h after the addition of ALA. Non-illuminated control plates were set up in parallel.

Correspondence: EK Matthews
Received 25 May 1994; revised 16 September 1994; accepted 30 September 1994

### Illumination

The 96-well plates were illuminated directly from below on a modified Sigma T2203 illuminator emitting white light at $\lambda > 400$ nm at an intensity of 0.2 mW $cm^{-2}$ or 0.47 mW $cm^{-2}$ for up to 16 min.

### MTT cell survival assay

Cell survival was measured using a rapid *in situ* spectrophotometric assay relying on the conversion of the yellow tetrazolium salt MTT to a blue-coloured formazan product, measuring intact mitochondrial dehydrogenase activity in living cells (Mossmann, 1983). Plates were assayed 24 h after illumination. MTT solution (3.75 mg $ml^{-1}$), 20 µl per well, was added under sterile conditions, incubated for 4 h at 37°C in a humidified atmosphere, and 100 µl per well of solubiliser [20% (w/v) sodium dodecyl sulphate (SDS) (Sigma) in 50% (v/v) dimethylformamide (DMF) (Fluka)/distilled water, pH 7.4 with 2.5% (v/v) glacial acetic acid/2.5% 1 N hydrochloric acid HCl] was added. The absorbance of each well was measured 24 h later in a Titer-Tek Multiskan (MCC 340) with dual absorbance reading at 540 nm and 690 nm.

### Porphyrin determination

Porphobilinogen (PBG) was measured using a colorimetric assay (Mauzerall and Granick, 1956) following a procedure described by Shedlofsky *et al.* (1987). Medium was removed from AR4-2J cell cultures grown in the absence or in the presence of 20 µM, 50 µM or 100 µM ALA, for either 24 h or 48 h, and discarded; the cell monolayer was rinsed once in phosphate-buffered saline (PBS) and the cells were scraped into 1 ml of 1.2 M perchloric acid and centrifuged at 250 *g* for 5 min. A 1 ml volume supernatant was then added to 1 ml of modified Ehrlich's reagent [1 g of *p*-dimethylaminobenzaldehyde (Sigma) in 30 ml of glacial acetic acid and 16 ml of 70% perchloric acid, diluted to 50 ml with glacial acetic acid] and the absorbance determined after 4 min at 595 nm. PBG content was determined from a standard curve of PBG (0.5–25 µM).

Cellular PPix concentration was determined spectrophotometrically (Henderson and Donovan, 1989). Cells were cultured in medium supplemented with ALA at 50 µM and 100 µM for 24 h, washed twice with PBS and removed with a rubber policeman. Cells were pelleted at 1700 r.p.m. for 10 min, resuspended in 5 ml of 0.1 N sodium hydroxide, sonicated and PPix absorbance determined at 430 nm. PPix concentration was deduced from a standard curve of PPix (1–50 µM). The protein content of the cells was determined by the Bio-Rad protein assay method, and PBG and PPix content expressed in terms of nmol $mg^{-1}$ protein.

### Singlet oxygen detection

$^1O_2$ was measured by bilirubin photo-oxidation (Diamond *et al.*, 1977), using 15 µM bilirubin in methanolic chloroform together with appropriate concentrations of the photosensitiser. Absorbance was determined at 453 nm on a Gilford 250 spectrophotometer and the results corrected for self-sensitised photo-oxidation of bilirubin.

### Statistical evaluation

All values quoted are means ± s.e.m. Statistical significance was determined by the two-tailed, unpaired Student's *t*-test. Values of $P < 0.05$ were considered statistically significant.

### Chemicals

ALA, PPix, PBG and bilirubin were all purchased from Sigma (Poole, Dorset, UK). PK11195 was a gift from Rhône-Poulenc Rorer. Ro5-4864, clonazepam and flumazenil were gifts from Roche.

### Results

### ALA phototoxicity

The survival of ALA-pretreated AR4-2J cells, as measured by the MTT cell survival assay, was found to decrease with increasing duration of illumination, as well as with increasing light intensity. Pretreatment of the serum-deprived AR4-2J cells with 10 µM ALA for 24 h elicited an illumination time-dependent phototoxicity (Figure 1). This time dependence was evident whether cells were grown in either serum-free or serum-containing medium. In other experiments, illumination of AR4-2J cells grown in serum-containing medium and pretreated with 10 µM ALA for 8 min, at 0.2 mW $cm^{-2}$, resulted in a <40% reduction in cell survival, i.e. to 62.0 ± 1.0% that of control values ($P < 0.05$, $n = 8$), whereas exposure to a light intensity of 0.47 mW $cm^{-2}$ for the same illumination time led to a more than 80% reduction in cell survival, i.e. to 16.0 ± 2.0% that of control values ($P < 0.005$, $n = 8$). Control values were defined by cell survival in the absence of both light and ALA.

The dose dependence of ALA-mediated phototoxicity was established in serum-free medium using an illumination time of 8 min, yielding an $EC_{50}$ of 6.6 ± 0.7 µM. There was a small decrease in the survival of non-illuminated control cells, but at the $EC_{50}$ of ALA for illuminated cells, there was still ⩾90% survival of the non-illuminated, control cells. Survival was again expressed as percentage survival in the absence of both light and ALA. Although in this series of experiments the cells were exposed to ALA for a standard time of 24 h before illumination, we have also observed in other experiments (not shown) that some photosensitivity of ALA-treated cells is evident even after only 4 h of ALA uptake.

### Cellular PBG content

The next step in the porphyrin biosynthetic pathway following ALA synthesis involves the formation of porphobilinogen (PBG) from the condensation of two molecules of ALA by ALA dehydratase (ALA-D). PBG was detected in AR4-2J cells incubated in ALA-supplemented medium (Figure 2). After 24 h exposure to 10 µM, 20 µM and 50 µM ALA, the PBG content of the AR4-2J cells increased with increasing dose of ALA; the increments were significant ($P < 0.01$) at 20 µM and 50 µM ALA. After 24 h, control PBG levels, in the absence of ALA, were 52.6 ± 5.3 nmol $mg^{-1}$ protein, which increased to 65.8 ± 8.6 nmol $mg^{-1}$ protein with 10 µM ALA, and further to 103.4 ± 10.3 nmol $mg^{-1}$ and 122.4 ± 11 nmol

**Figure 1** Time dependence of ALA-mediated phototoxicity. The effect of ALA 10 µM on cell survival following exposure to light at 0.2 mW $cm^{-2}$. Survival is expressed as a percentage of cells receiving neither light nor ALA. Values are means ± s.e.m. ($n = 6$); where no error bars are seen they lie within the symbol.

$mg^{-1}$ after exposure to 20 μM and 50 μM ALA respectively. After 48 h pre-exposure to ALA there was a small amount of PBG accumulation, but this was far less than that seen after 24 h. After 48 h incubation in the absence of ALA, the PBG concentration was only 20.0 ± 2.5 nmol $mg^{-1}$ protein. Addition of 20 μM and 50 μM ALA caused a significant increase in the PBG content to 27.0 ± 0.8 nmol $mg^{-1}$ protein ($P < 0.05$) and 37.5 ± 4.5 nmol $mg^{-1}$ protein ($P < 0.01$) respectively.

*Cellular PPix*

Following measurement of the intermediary pyrrole, PBG, in porphyrin biosynthesis, the final porphyrin in the pathway, PPix, was measured using a spectrophotometric assay. The PPix content of AR4-2J cells cultured in the presence of 50 μM and 100 μM ALA for 24 h was determined (Figure 3). In the absence of ALA the PPix content was 2.5 ± 1.1 nmol $mg^{-1}$ protein increasing significantly ($P < 0.01$) to 5.0 ± 1.6 nmol $mg^{-1}$ protein with 50 μM ALA, and further to 9.7 ± 3.8 nmol $mg^{-1}$ protein ($P < 0.001$) with 100 μM ALA.

**Figure 2** PBG content of AR4-2J cells following ALA administration. PBG accumulation in AR4-2J cells following exposure to 10 μM, 20 μM and 50 μM ALA for 24 h (□) and for 48 h (■). Values are means ± s.e.m. for three separate experiments. *$P < 0.05$ and **$P < 0.01$. Control cells were incubated in serum-free medium in the absence of ALA.

**Figure 3** PPix content of AR4-2J cells following ALA administration. PPix accumulation in AR4-2J cells following exposure to 50 μM and 100 μM ALA for 24 h. Values are means ± s.e.m. ($n = 6$). **$P < 0.01$ and ***$P < 0.005$.

*Singlet oxygen generation*

Photodynamic therapy is believed to exert its tumoricidal effect through generation of singlet oxygen ($^1O_2$), a short-lived excited state of molecular oxygen, that can cause cytolysis (Weishaupt *et al.*, 1976). Currently, the primary sites of photodynamic attack by $^1O_2$ are thought to be cellular and mitochondrial membranes, but nucleic acids and proteins are also susceptible to photo-oxidative action (Foote, 1984). The ability of the photodynamic agent ALA to generate $^1O_2$, as detected by bilirubin photo-oxidation assay, was investigated along with the porphyrin product of ALA metabolism, PPix (Figure 4). ALA at concentrations of 1 μM and 100 μM showed no capacity to generate $^1O_2$. PPix, 1 μM, however, did cause bilirubin photo-oxidation on exposure to light, an indication of $^1O_2$ generation. This is evidence that the PPix produced by treatment of AR4-2J cells with exogenous ALA is responsible for the oxidative photodamage that occurs upon irradiation of the cells with light, rather than being dependent on the ALA itself. The $^1O_2$ generation profile of tetrasulphonated aluminium phthalocyanine ($AlPcS_4$) was included as a positive control in the experiment as it is an efficient photosensitiser with a high quantum yield for $^1O_2$ ($\varphi = 0.4$). At equimolar concentrations $ALPcS_4$ generates $^1O_2$ more efficiently than PPix.

*Effects of benzodiazepine compounds on ALA-mediated phototoxicity*

PPix has been identified as an endogenous ligand of high affinity for the mitochondrial benzodiazepine receptor (MBR) (Verma *et al.*, 1987), a binding site which may mediate a wide range of effects on cellular respiration (Hirsch *et al.*, 1988) and cellular proliferation (Stepien *et al.*, 1991). The MBR has also been implicated as a site for PPix and haem translocation across mitochondrial membranes (Anholt, 1986). The ability of both centrally and mitochondrially acting benzodiazepine compounds to interfere with phototoxicity elicited through ALA-induced PPix production in AR4-2J cells was therefore assessed (Figure 5). In all these experiments an $EC_{50}$ value was determined for each individual experiment and statistical significance calculated as the difference between the mean values obtained for each set of experiments. Clonazepam (CLO) had no significant effect on

**Figure 4** Photosensitiser generation of singlet oxygen. Control $^1O_2$ generation of bilirubin is shown in open circles (○). ALA 1 μM (■) and 100 μM (□). PPix 1 μM (▲) and $AlPcS_4$ 1 μM (●), upon illumination at 0.2 mW $cm^{-2}$ for up to 30 min. All values are corrected for self-oxidation of bilirubin.

ALA-mediated phototoxicity (Figure 5a), the $EC_{50}$ of phototoxicity for ALA alone, 6.6 ± 0.7 μM, being increased minimally to 6.8 ± 0.8 μM and 7.4 ± 0.9 μM in the presence of 10 μM and 20 μM CLO, respectively. Likewise, with flumazenil (FLU) there was no significant increase in $EC_{50}$, i.e. from 6.6 ± 0.7 μM to only 7.3 ± 1.0 μM at both concentrations, i.e. 10 μM and 20 μM. CLO and FLU are centrally acting benzodiazepines with very low affinities for the MBR. In contrast, the two most potent peripherally acting compounds known (those used here), are the isoquinoline carboxamide PK11195 and the benzodiazepine-2-one Ro 5-4864. Both compounds affected ALA-mediated phototoxicity markedly (Figure 5c and d). In the presence of 10 μM PK11195, the $EC_{50}$ for phototoxicity doubled from 6.6 ± 0.7 μM to 15.2 ± 4.5 μM, and with 20 μM PK11195 the $EC_{50}$ was further increased to 19.5 ± 0.9 μM ($P<0.001$, $n = 5$). Likewise, pretreatment of the AR4-2J cells with Ro 5-4864 altered considerably the $EC_{50}$ for ALA-mediated phototoxicity. In the presence of 10 μM Ro 5-4864, the $EC_{50}$ increased from 6.6 ± 0.7 μM to 16.4 ± 2.3 μM ($P<0.002$, $n = 6$), and further to 26.7 ± 2.5 μM in the presence of 20 μM Ro 5-4864 ($P<0.0005$, $n = 6$). Although major effects were found on ALA-mediated phototoxicity, the benzodiazepines may have some direct action also on cell survival in the absence of ALA. Thus, the centrally acting ligand CLO caused an 8 ± 1% and 10 ± 1% decrease in cell survival at 10 μM and 20 μM ($P<0.01$, $n = 6$), respectively; similarly with FLU the decrease was 15 ± 1% and 16 ± 1% at 10 μM and 20 μM ($P<0.01$, $n = 6$) respectively. PK11195 also produced a small decrease in cell survival, of 13 ± 1% and 17 ± 1% at 10 μM and 20 μM ($P<0.01$, $n = 6$) respectively, whereas Ro 5-4864, diminished cell survival by 15 ± 0.5% at 10 μM ($P<0.01$, $n = 6$) and by 30 ± 1% at 20 μM ($P<0.005$, $n = 6$).

### Discussion

The present results demonstrate clearly that rat pancreatic tumour AR4-2J cells can be inactivated by light following exposure to an ALA-enriched culture medium for 24 h. This photoinactivation is mediated by the photodynamic effects of endogenous porphyrins, in particular PPix, and is dependent on the light intensity, duration of illumination and concentration of precursor, ALA. Although the results described here show the effects following 24 h exposure to ALA, we have also noted that photodynamic effects can be observed after as little as 4 h exposure to ALA. This is consistent with the findings of *in vivo* studies in which there was measurable porphyrin accumulation and photodynamic action evident 3–4 h after exposure to ALA in tumours of stomach, colon and bladder mucosa (Bedwell *et al.*, 1992; Loh *et al.*, 1993).

ALA is an essential component of porphyrin biosynthesis in all cells. The intramitochondrial generation of ALA by ALA synthase (ALA-S) is the rate-limiting step normally repressed by the end-product haem. ALA is condensed by

**Figure 5** Effects of benzodiazepines on ALA-mediated phototoxicity. **a**, The effects of clonazepam (CLO) on ALA-mediated phototoxicity: ALA alone (○), or in the presence of CLO 10 μM (●) or CLO 20 μM (Δ). **b**, The effects of flumazenil (FLU): ALA alone (○) or in the presence of FLU 10 μM (●) or FLU 20 μM (Δ). **c**, The effects of PK11195: ALA alone (○) or in the presence of PK 10 μM (●) and PK 20 μM (Δ). **d**, The effects of Ro 5-4864: ALA alone (○) or in the presence of Ro 10 μM (●) or Ro 20 μM (Δ). Values are weighted means ± s.e.m. ($n = 5$ for **a**, **b** and **c**; $n = 6$ for **d**); significance determined from original data. Where no error bars are seen they lie within the symbol.

ALA dehydratase (ALA-D) to yield the pyrrole porphobilinogen (PBG), deamination of which to uroporphyrinogen by PBG deaminase (PBG-D) is thought to be a secondary rate-limiting step, and overactivity of which may lead to an excess of photosensitising prophyrin products. PPix is formed by successive decarboxylation and re-entry into the mitochondrion of the intermediary porphyrinogens. Ultimately haem is formed by the insertion of ferrous iron. The enzyme responsible for the incorporation of iron into PPix and the formation of haem is ferrochelatase, a deficiency of which will result in the accumulation of photoactive PPix and cellular photodamage. In fact, not only has a low ferrochelatase activity been recognised in malignant cells (Rubino and Rasetti, 1966; van Hillegersberg *et al.*, 1992), but, in the more recent study, the activity of PBG-D, the enzyme responsible for the deamination of PBG to uroporphyrinogen, was also shown to be elevated. These reciprocal changes in enzyme activity may together lead to a considerably enhanced accumulation of photoactive porphyrins in tumour cells, making them particularly susceptible to photolysis and hence a prime target for photodynamic therapy.

Both the intermediary metabolite of porphyrin biosynthesis, PBG, and the end-product, PPix, have been identified in rat pancreatic tumour cells of the AR4-2J cell-line used in this study. Upon supplementation of the culture medium with ALA, AR4-2J cells accumulate the pyrrole intermediate, PBG, and the photoactive porphyrin, PPix. The accumulation of PBG in the cells was most marked after incubation for 24 h in the presence of an ALA-enriched medium. After exposure to ALA for 48 h, the control levels of PBG were significantly less than those seen after 24 h. This may be attributable to utilisation of the PBG in the porphyrin synthetic pathway to form other porphyrin products, or to transamination of some ALA to α-ketoglutarate; it may also be due, in part, to lower cell viability after culture for 48 h in the absence of serum. Furthermore, PPix accumulation in AR4-2J cells was evident following a 24 h treatment with ALA, the PPix content of the cells increasing with increasing doses of ALA.

Photodynamic cytolysis can be attributed predominantly to the highly reactive molecular oxygen species, $^1O_2$. ALA itself over a wide range of concentrations, i.e. 1 μM to 100 μM, does not generate $^1O_2$. The main product of porphyrin biosynthesis, PPix, is however a very effective generator of $^1O_2$. Light-activated PPix has been shown to exert its lethal effects on neoplastic cells by generation not only of $^1O_2$, but also of hydroxyl ($OH^{\cdot -}$) radicals (Kessel, 1977), and the cellular effects of PPix as a photosensitiser *per se* are well documented. For example, PPix has been shown to have a photodynamic action on isolated rat liver mitochondria, leading to uncoupling and inhibition of oxidative phosphorylation, energy dissipation, inhibition of respiration, and swelling and disruption of mitochondria (Sandberg and Romslo, 1980). Photoactivated PPix will also destroy Friend erythroleukaemia, myelocytic leukaemia, Burkitt lymphoma and mastocytoma cells (Malik and Djaldetti, 1980).

PPix binds to the mitochondrial benzodiazepine receptor (MBR) with high affinity, the $K_i$ for displacement of benzodiazepine ligands being 15 nM (Verma *et al.*, 1987). The MBR is associated with (i) a voltage-dependent anion channel (VDAC) similar to mitochondrial porin, which permits the transport of metabolites between mitochondria and cytoplasm, (ii) an adenine nucleotide translocator (ANT) providing a physical path for cytoplasmic ADP exchange with mitochondrial ATP and (iii) a number of other proteins and enzymes including hexokinase and creatine kinase. The mitochondrial benzodiazepine recognition site is located on the outer mitochondrial membrane, and the MBR complex with VDAC, ANT and the kinases is organised at contact sites between the outer and inner mitochondrial membranes. Thus, the MBR may mediate a wide range of physiological effects including respiratory control and cellular proliferation. We have clearly demonstrated in this study that ligands such as the isoquinoline carboxamide PK11195 and the benzodiazepine Ro 5-4864 will also affect the phototoxic response elicited by exogenous ALA in AR4-2J pancreatoma cells. Both PK11195 and Ro 5-4864 markedly increase the $EC_{50}$ for ALA-mediated phototoxicity, thus exerting a photoprotective effect, with cell survival being increased from <10% to >90%. This effect is specific to the mitochondrially active benzodiazepine ligands because the effects were not replicated by the centrally active ligands clonazepam and flumazenil. Furthermore, it is known that neither ALA nor PBG has any affinity for the MBR (Verma and Snyder, 1988).

How then do PK11195 and Ro 5-4864 produce their photoprotective action? They may act competitively and oppose the binding of PPix, produced from the exogenous ALA, to the MBR, thereby inhibiting the translocation of PPix from the mitochondria to the cytoplasmic compartment, or vice versa, and so restrict the locus of photodamage elicited upon light activation. It is also conceivable that the ligands may interfere with the production of PPix from its precursors, since the precursors to porphyrin must traverse the mitochondrial membrane in order for PPix to be formed. PK11195 and Ro 5-4864 are believed to bind to overlapping, but not identical, sites on the MBR. A small sequence near the C-terminal end of the MBR molecule may be involved in the binding of Ro 5-4864, but not of PK11195 (Farges *et al.*, 1993). This small spatial and molecular difference in binding site may account for the slight difference in the degree of photoprotection conferred by the two ligands, Ro 5-4864 exerting a greater photoprotective effect than PK11195 at 10 μM and 20 μM.

In view of these observations it is interesting to note that Kessel (1988), using mesoporphyrin and haematoporphyrin derivative (HpD), found no inhibition of porphyrin binding to isolated rat kidney mitochondria by the benzodiazepines, nor were acute effects of the MBR ligands detected on the photosensitisation of intact cells, although these experiments with exogenous porphyrins were of comparatively short duration. Our results, involving lower concentrations of drug present for more extended time periods, indicate that in intact cells it is the mitochondrial translocation of the endogenous porphyrins, including PPix, which is likely to be primarily affected by MBR ligands. The MBR is an important mediator of cellular processes in tumour cells, activation of which by ligands such as Ro 5-4864 has been implicated in antiproliferation of thymoma cell lines (Wang *et al.*, 1984*b*). There are recent reports also of an increase in the number of MBR binding sites in colonic adenocarcinoma tissue, to 3.4 times that of normal tissue (Katz *et al.*, 1988), and in ovarian neoplasms 3–5 times that of normal or benign tissue (Katz *et al.*, 1990). Activation of the MBR by the endogenous ligand, PPix, may therefore play an important role in tumour cell metabolism *per se*. PPix itself has also been shown to be antiproliferative in mouse spleen lymphocytes (Stepien *et al.*, 1991).

In conclusion, we have established that pancreatoma cells become sensitised to light even after quite short periods of ALA uptake. This points to a rapid increase in the rate of PPix synthesis in exocrine tumour cells, a fact that we have been able to confirm by measurement of cellular PBG and PPix following ALA treatment. Finally, this study not only highlights the role that the mitochondrial benzodiazepine receptor may play in ALA-mediated phototoxicity, but it demonstrates how ligands for the MBR may be used deliberately to interfere with the actions of PPix, eliciting a protective effect when cells pretreated with the ligand are exposed to both ALA and light. These drugs, which are devoid of central actions, therefore have the potential to rescue cells from porphyrin-induced photolysis and may prove to be of use clinically for this purpose.

**Acknowledgements**

SLR holds an MRC studentship. We are grateful to Mrs Margaret Forsyth for expert technical assistance.

### References

ANHOLT R. (1986). Mitochondrial benzodiazepine receptors as potential modulators of intermediary metabolism. *Trends Pharmacol. Sci.*, **7**, 506-511.

BEDWELL J, MACROBERT AJ, PHILLIPS D AND BOWN SG. (1992). Fluorescence distribution and photodynamic effect of ALA-induced PPIX in the DMH rat colonic tumour model. *Br. J. Cancer*, **65**, 818-824.

DIAMOND I, GRANELLI SG AND McDONAGH AF. (1977). Photochemotherapy and photodynamic toxicity: simple methods for identifying potentially active agents. *Biochem. Med.*, **17**, 121-127.

FARGES R, JOSEPH-LIAUZUN E, SHIRE D, CAPUT D, LE FUR G, LOISON G AND FERRARA P. (1993). Molecular basis for the different binding properties of benzodiazepines to human and bovine peripheral-type benzodiazepine receptors. *FEBS Lett.*, **335**, 305-308.

FOOTE CS. (1984). Mechanisms of photooxygenation. In *Porphyrin Localization and Treatment of Tumours*, DR Doiron and CJ Gomer (eds) pp. 3-18. Alan R Liss: New York.

HENDERSON BW AND DONOVAN JM. (1989). Release of prostaglandin $E_2$ from cells by photodynamic treatment *in vitro*. *Cancer Res.*, **49**, 6896-6900.

HIRSCH JD, BEYER C, MALOWITZ L, BEER B AND BLUME A. (1988). Mitochondrial benzodiazepine receptors mediate inhibition of mitochondrial respiratory control. *Mol. Pharmacol.*, **34**, 157-163.

KATZ Y, EITAN A, AMIRI Z AND GARISCH M. (1988). Dramatic increase in peripheral benzodiazepine binding sites in human colonic adenocarcinoma as compared to normal colon. *Eur. J. Pharmacol.*, **148**, 483-484.

KATZ Y, BEN-BARUCH G, KLOOG Y, MENCZER J AND GARISCH M. (1990). Increased density of peripheral benzodiazepine-binding sites in ovarian carcinomas as compared with benign ovarian tumours and normal ovaries. *Clin. Sci.*, **78**, 155-158.

KESSEL D. (1977). Effects of photoactivated porphyrins at the cell surface of leukemia L1210 cells. *Biochemistry*, **16**, 3443-3449.

KESSEL D. (1988). Interactions between porphyrins and mitochondrial benzodiazepine receptors. *Cancer Lett.*, **39**, 193-198.

LOH CS, MACROBERT AJ, BEDWELL J, REGULA J, KRASNER N AND BOWN SG. (1993). Oral versus intravenous administration of 5-aminolaevulinic acid for photodynamic therapy. *Br. J. Cancer*, **68**, 41-51.

LONGNECKER DS, LILJA HS, FRENCH J, KUHLMANN E AND NOLL W. (1979). Transplantation of azarserine-induced carcinomas of the pancreas in rats. *Cancer Lett.*, **7**, 197-202.

MALIK Z AND DJALDETTI M. (1980). Destruction of erythroleukemia, myelocytic leukemia and Burkitt lymphoma cells by photoactivated protoporphyrin. *Int. J. Cancer*, **26**, 495-500.

MALIK Z AND LUGACI H. (1987). Destruction of erythroleukaemic cells by photoactivation of endogenous porphyrins. *Br. J. Cancer*, **56**, 589-595.

MAUZERALL D AND GRANICK S. (1956). The occurrence and determination of δ-aminolaevulinic acid and porphobilinogen in urine. *J. Biol. Chem.*, **219**, 435-446.

MOSSMAN T. (1983). Rapid colorimetric assay for cellular growth and survival: application to proliferation and cytotoxic assays. *J. Immunol. Methods*, **65**, 55-63.

POTTIER RH, CHOW Y, LAPLANTE J, TRUSCOTT TG AND KENNEDY JC. (1986). Noninvasive technique for obtaining fluorescence excitation and emission spectra *in vivo*. *Photochem. Photobiol.*, **44**, 679.

RATCLIFFE SL AND MATTHEWS EK. (1994). The photodynamic actions of δ-aminolaevulinic acid on rat pancreatoma AR4-2J cells. *Br. J. Pharmacol.*, **112**, 141P.

RUBINO GF AND RASETTI L. (1966). Porphyrin metabolism in human neoplastic tissues. *Panminerva Medica*, **8**, 290-292.

SANDBERG S AND ROMSLO I. (1980). Porphyrin-sensitized photodynamic damage of isolated rat liver mitochondria. *Biochim. Biophys. Acta*, **593**, 187-195.

SHEDLOFSKY SI, SINCLAIR PR, BONKOVSKY HL, HEALEY JF, SWIM AT AND ROBINSON JM. (1987). Haem synthesis from exogenous 5-aminolaevulinate in cultured chick-embryo hepatocytes - effect of inducers of cytochromes P-450. *Biochem. J.*, **248**, 229-236.

STEPIEN E, KUNERT-RADEK J, STANISZ A, ZEREK-MELEN G AND PAWLIKOWSKI M. (1991). Inhibitory effect of porphyrins on the proliferation of mouse spleen lymphocytes *in vitro*. *Biochem. Biophys. Res. Comm.*, **174**, 313-322.

VAN HILLEGERSBERG R, VANDENBERG JWO, KORT WJ, TERPSTRA OT AND WILSON JHP. (1992). Selective accumulation of endogenously produced porphyrins in a liver metastasis model in rats. *Gastroenterology*, **103**, 647-651.

VERMA A AND SNYDER SH. (1988). Characterization of porphyrin interactions with peripheral type benzodiazepine receptors. *Mol. Pharmacol.*, **34**, 800-805.

VERMA A, NYE JS AND SNYDER SH. (1987). Porphyrins are endogenous ligands for the mitochondrial (peripheral-type) benzodiazepine receptor. *Proc. Natl Acad. Sci. USA*, **84**, 2256-2260.

WANG J, MORGAN J AND SPECTOR S. (1984*a*). Differentiation of Friend erythroleukemia cells induced by benzodiazepines. *Proc. Natl Acad. Sci. USA*, **81**, 3770-3772.

WANG J, MORGAN J AND SPECTOR S. (1984*b*). Benzodiazepines that bind at peripheral sites inhibit cell proliferation. *Proc. Natl Acad. Sci. USA*, **81**, 753-756.

WEISHAUPT KR, GOMER CJ AND DOUGHERTY TJ. (1976). Identification of singlet oxygen as the cytotoxic agent in photoinactivation of a mouse tumour. *Cancer Res.*, **36**, 2326-2329.